# Transcriptome Atlas of 16 Donkey Tissues

**DOI:** 10.3389/fgene.2021.682734

**Published:** 2021-08-09

**Authors:** Yinan Wang, Xinyao Miao, Zicheng Zhao, Yonghui Wang, Shuaicheng Li, Changfa Wang

**Affiliations:** ^1^Liaocheng Research Institute of Donkey High-Efficiency Breeding and Ecological Feeding, Liaocheng University, Liaocheng, China; ^2^College of Forensic Medicine, Xi’an Jiaotong University, Xi’an, China; ^3^Department of Computer Science, City University of Hong Kong, Hong Kong, China; ^4^Shenzhen Byoryn Technology Co., Ltd, Shenzhen, China

**Keywords:** donkey, RNA sequencing, proteomics, iBAQ, lysozyme

## Abstract

Donkeys (*Equus asinus*) are important livestock with great economic value in meat, skin, and milk production. However, a lack of knowledge of the transcriptome landscape across a wide range of donkey tissues limits genetic selective breeding and conservation. Here we used transcriptomics to describe the transcriptome landscape, classify the tissue-specific gene expression across all primary donkey tissues, and present supplementary analyses on the protein level of additional donkey milk samples. Overall, 16,013 protein-coding genes and 21,983 transcripts were mapped to the reference genome, including 6,778 ubiquitously expressed genes and 2,601 tissue-enriched genes. Functional analysis revealed that the function of the tissue-enriched genes was highly tissue specific. Tissue-elevated genes that could be associated with unique phenotypes in donkey were analyzed. The results showed that, compared with those in human and other livestock, the lysozyme gene in donkey breast was specifically and highly expressed. The calcium-binding lysozyme, encoded by the lysozyme gene, was also detected in high amounts in donkey milk. Given those intact lysozyme genes that predict potentially functional calcium-binding lysozyme found in only a few species (e.g., donkey and horse), the high expression of the lysozyme gene in donkey breast may contribute to the high lysozyme content in donkey milk. Furthermore, 71% of the proteins in donkey milk overlapped with human milk protein, higher than the overlapping rates of bovine, sheep, and swine with humans. The donkey transcriptomic resource contributes to the available genomic resources to interpret the molecular mechanisms underlying phenotype traits.

## Introduction

Donkeys (*Equus asinus*) are one of the most common species in agricultural production and transportation (Polidori et al., 2015), with considerable commercial and economic value in meat, skin, and milk production (Camillo et al., 2018; Zhang et al., 2020). Although donkeys and horses belong to a single genus, donkeys have unique genetic traits, such as heat and drought tolerance. In 2019, the global donkey population was estimated at 50 million, among which the domestic donkey accounts for 61% in Africa and 26% in Asia. The six leading countries in donkey breeding are Ethiopia (8.7 million), Sudan (7.6 million), Pakistan (5.4 million), Chad (3.6 million), Mexico (3.2 million), and China (2.6 million; FAOSTAT database).^1^ In Asia, donkey skin and meat contributed to food and healthy productions (Camillo et al., 2018; Zhang et al., 2020). Donkey meat has higher crude protein, essential amino acids, and unsaturated fatty acids and lower total fat, cholesterol, and calories than beef and mutton meat (Marino et al., 2015). Donkey milk has become increasingly attractive due to its ingredients and medicinal value, such as antimicrobial, antiviral, anti-inflammatory, antiproliferative (Aspri et al., 2017; Vincenzetti et al., 2017), and antioxidant (Martini et al., 2018) activities. China produces approximately 0.27 million tons of donkey milk annually (Luo et al., 2019) and has a long history of alimentary and medicinal usage of donkey milk (Li et al., 2018).

With the ultimate goal of interpreting the molecular mechanisms underlying the phenotype of economic value and increasing the rate of genetic gain through artificial selection, much interest has been given to annotating livestock genes at the levels of DNA (Jónsson et al., 2014; Ai et al., 2015; Chen et al., 2018), RNA (Jiang et al., 2014; Sun et al., 2019), and protein (Roncada et al., 2012; D’Alessandro and Zolla, 2013). To date, genomic studies assembling a subchromosome-level donkey genome laid the foundation for genetic improvement or manipulation (Renaud et al., 2018). Untargeted proteomics disclosed the antibacterial potential of donkey milk (Spada et al., 2021). Although donkeys were preferred over other equines because of their affordability, survivability, and medical and economic value, a lack of knowledge of the transcriptome landscape across a wide range of donkey tissues limits genetic selective breeding and conservation. The systematic and comprehensive transcriptomic profiles of donkeys are crucial to identify tissue-specific genes and the phenotypes or functions that these genes contribute. They could also provide a deeper understanding of the mechanisms of the genetic traits associated with cells, tissues, and organs in donkey.

A chromosome-level reference genome of Dezhou donkey was recently assembled (Wang et al., 2020), providing the best contiguity and the most comprehensive annotation by far. The transcriptome data of 13 tissues (17 samples) were used to assist with genome assembly, and the similarities and differences in gene functions between horse and donkey were analyzed. In this work, the tissue types were increased from 13 to 17 and the sample size from 17 subjects to 54 subjects to ensure that each tissue had at least three biological replicates, with the exception of the ovary. In addition, 16 tissues were used for RNA sequencing, and donkey milk was used for proteome quantification. A more in-depth transcriptomic study of the 16 different tissues was performed to construct a genome-wide gene expression profile of donkey tissues, in particular, tissue-elevated genes that may be associated with the unique phenotypes in donkey. The lysozyme gene (*LYZ*) attracted the attention of researchers, as it was highly expressed in the breast than in other tissue samples. Subsequently, the high levels of calcium-binding lysozyme (LYSC1), which was encoded by *LYZ*, in donkey milk were validated using quantitative mass spectrometry. The increased expression of *LYZ* in donkey breast may contribute to the high LYSC1 content in donkey milk.

## Materials and Methods

### Animal Material

A total of 46 samples from 16 different tissues (Table 1) of 10 Dezhou donkeys were collected for RNA sequencing. Eight milk samples (45 ml/sample) from eight other Dezhou donkeys were also collected for quantitative proteomics. Ten donkeys were anesthetized using pentobarbital (100 mg/kg, i.v.) and euthanized by exsanguination. The tissue samples were chopped to small sections and thoroughly washed using DNase-free and RNase-free water. They were placed in liquid nitrogen to freeze and then stored at −20°C. The milk samples were frozen immediately after collection at each milking and stored at −20°C until analysis. A detailed sample information is provided in Supplementary Table 1. The donkeys were utilized in this study in line with protocols of care and use of laboratory animals, following receipt of permission from the Animal Care and Use Committee of Shandong Academy of Agricultural Sciences.

**TABLE 1:** Sample information.

Donkey	Gender	Age (months)	Number of tissues	Tissue type	Experiment
Donkey1	Male	20	13	Heart, kidney, liver, lung, brain, spleen, stomach, blood, cecum, skin, muscle, testis, epididymis	RNA sequencing
Donkey2	Male	24	3	Skin, muscle, cecum	RNA sequencing
Donkey3	Male	18	3	Skin, muscle, cecum	RNA sequencing
Donkey4	Male	21	8	Heart, kidney, liver, lung, brain, spleen, stomach, blood	RNA sequencing
Donkey5	Male	24	8	Heart, kidney, liver, lung, brain, spleen, stomach, blood	RNA sequencing
Donkey6	Male	25	2	Testis, epididymis	RNA sequencing
Donkey7	Male	19	2	Testis, epididymis	RNA sequencing
Donkey8	Female	48	3	Chestnut, breast, ovary	RNA sequencing
Donkey9	Female	50	2	Chestnut, breast	RNA sequencing
Donkey10	Female	44	2	Chestnut, breast	RNA sequencing
Donkey11	Female	49	1	Donkey milk	Protein quantitation
Donkey12	Female	45	1	Donkey milk	Protein quantitation
Donkey13	Female	70	1	Donkey milk	Protein quantitation
Donkey14	Female	62	1	Donkey milk	Protein quantitation
Donkey15	Female	49	1	Donkey milk	Protein quantitation
Donkey16	Female	85	1	Donkey milk	Protein quantitation
Donkey17	Female	84	1	Donkey milk	Protein quantitation
Donkey18	Female	51	1	Donkey milk	Protein quantitation

### RNA Sequencing

The procedures for RNA sequencing were adopted from a previous study (Wang et al., 2020). In brief, RNA was obtained using TRIzol (Thermo Fisher Scientific, CA, United States) and evaluated with an Agilent 2100 Bioanalyzer (Agilent Technologies, CA, United States). After screening, high-quality RNA was sequenced (RNA integrity number >7.0 and 28S/18S ratio > 1.0). The total RNA was purified using oligo-dT beads. Thereafter, the mRNA strands were fragmented using Fragmentation Buffer (Ambion, Thermo Fisher Scientific, CA, United States). The cDNA libraries were then constructed using the templates. The cDNA was sequenced on the Illumina HiSeq 4000 system for paired-end reads with a read length of 100 bases.

### Gene Expression and Specificity Classification

RNA sequencing data was aligned by utilizing HISAT2 software (v2.1.0) (Kim et al., 2019) based on donkey reference sequences (EquAsi1.0, GenBank assembly accession: GCA_016077325.1) as previously described (Wang et al., 2020). StringTie (v2.1.4) was used for transcript assembly and quantification, including fragments per kilobase million (FPKM) and transcripts per million (TPM; Pertea et al., 2015). Novel transcripts and genes were predicted using gffcompare (v0.11.2) (Pertea and Pertea, 2020). The transcripts whose class code was annotated by “u,” length >200 nt, and exon number >1 were retained and subjected to novel gene annotation. DIAMOND, kofamscan, and hmmer were used to annotate novel genes on the basis of five public databases, namely, NCBInr,^2^ SwissProt,^3^ eggNOG,^4^ Kyoto Encyclopedia of Genes and Genomes (KEGG)^5^, and Pfam,^6^ with >500 bit score, >50% pident, and >1e-20 *E*-value. Further studies were performed in accordance with the reference genes. In order to compare the donkey mRNA profile with human or other livestock data, we used the classification scheme of Uhlén et al., which was previously developed for human mRNA profiling. The gene and transcript expression levels were estimated based on the average FPKM of each tissue sample (Uhlén et al., 2015). It ratifies the genes into six classes: “not detected” (FPKM <1 in all tissues), “tissue enriched” (fivefold higher FPKM than any other tissue), “group-enriched” (fivefold higher average FPKM in two to seven tissues, relative to the rest of tissues), “expressed in all” (FPKM >1 in all tissues), “tissue-enhanced” (fivefold FPKM higher than the average FPKM of the rest of tissues), and “mixed” (did not match the aforementioned categories).

### Principal Component Analysis and Hierarchical Clustering

The principal component analysis (PCA) and hierarchical clustering analysis of individual samples were performed on the TPM for all genes. PCA was generated using the R (v4.0.3) function “prcomp.” In unsupervised hierarchical clustering, the Spearman correlation-based distance for each sample and the average linkage method were used. The clusters were displayed on a heat map developed based on the pairwise correlation coefficients. Heat maps were plotted with the pheatmap R package.

### Network Analysis

The Cytoscape 3.0 software was adopted to create a network of tissue- and group-enriched genes which were classified on the basis of FPKM (Shannon et al., 2003). The network included the group-enriched nodes with a maximum of five connections and at least three expressed genes. FPKM was used as a measure of gene expression.

### Functional Enrichment Analysis

Gene Ontology (GO) and pathway enrichment analysis of tissue-enriched and ubiquitously expressed genes, classified on the basis of FPKM, were conducted on ClusterProfiler package (v3.18.0) in R software. *P*-values were adjusted by false discovery rate (Yu et al., 2012). In accordance with the functional annotation of Uniprot (see text footnote 3), the related protein products of the genes were classified into categories of predicted intracellular proteins, predicted secreted proteins, and genes with isoforms belonging to both categories.

### Protein Preparation

The milk samples in lysis buffer (a mixture of 2 mM EDTA, 1 mM PMSF, 40 mM Tris–HCl, pH 8.5, 4% CHAPS, 2 M thiourea, and 7 M urea) were agitated on ice. Subsequently, the extracted proteins were incubated at 56°C with 10 mM dithiothreitol (final concentration) for 1 h to decrease the number of disulfide bonds. The milk was then alkylated for 1 h in a dark room using 55 mM IAM (final concentration). This mixture was place on chilled acetone (at −20°C), 4 × volume of the milk, overnight to allow precipitation. It was then centrifuged at 30,000 × *g* under 4°C to form a protein pellet that was reconstituted in 0.5 M TEAB (Applied Biosystems, Milan, Italy) and agitated on ice. The suspension was then centrifuged at 30,000 × *g* under 4°C, and the protein was quantified with the Bradford assay.

### iTRAQ Labeling

The total protein (100 μg) from each milk sample was heated at 37°C for 16 h using Trypsin Gold (Promega, Madison, WI, United States). The digestion was done at a ratio of 1:20, i.e., trypsin to protein. Thereafter, the protein digests were dried in vacuum through centrifugation. The peptide cocktails were suspended in 0.5 M TEAB and then treated with 8-plex iTRAQ reagent (Applied Biosystems), following the protocols of the manufacturer.

### HPLC

The labeled peptide cocktail was first fractioned on the LC-20AB HPLC Pump system (Shimadzu, Kyoto, Japan) and thereafter suspended in 4 ml buffer A mixture (25 mM NaH_2_PO_4_ in 25% ACN, pH 2.7). The peptides were loaded onto a 4.6 × 250-mm Ultremex SCX column mixed with 5-μm particles (Phenomenex). Elution was performed at 1 ml/min for 10 min using buffer A. The second elusion was performed for 27 min using 5–60% buffer B (25 mM NaH_2_PO_4_ and 1 M KCl in 25% ACN, pH 2.7) before the final elusion for 1 min using 60–100% buffer B. The peptides were added into 100% buffer B and allowed to incubate for 1 min before equilibrating using buffer A for 10 min prior to the next injection. The absorbance of the peptide cocktail was measured regularly at 214 nm to monitor the progress of elution. Samples were also obtained at 1 min each. Finally, 20 fractions of the peptides were pooled together before desalting using Strata X C18 column (Phenomenex). The peptides were then dried in vacuum.

### LC-ESI-Tandem Mass Spectrometry

The peptide extracts were resuspended in buffer A (a mixture of 2% CAN and 0.1% FA) before centrifugation at 20,000 × *g* for 10 min. Thereafter, 10 μl of the supernatant was added onto an LC-20AD nano HPLC (Shimadzu, Kyoto, Japan) and eluted. Loading of samples was performed at the rate of 8 μl/min for 4 min before running at 300 nl/min for 44 min. The concentration of B (a mixture of 98% CAN and 0.1% FA) started from 2 to 35% and thereafter at 80% for 2 min, at 80% B for 4 min, and finally at 5% for 1 min.

Nanoelectrospray ionization of the peptides was performed before the tandem mass spectrometry (MS). The latter process was performed in Q EXACTIVE HPLC (Thermo Fisher Scientific, San Jose, CA, United States). An Orbitrap operating at a resolution of 70,000 was employed to identify unbroken peptides. Ion fragments were detected using an Orbitrap at a resolution of 17,500. The voltage for electrospray was 1.6 kV. The automatic gain control target for full MS and MS2 were 3e6 and 1e5, respectively. The m/z scan range of the MS scans was 350–2,000 Da and 100–1,800 m/z for MS2 scans.

### Quantification and Characterization of Proteome

Raw data generated from the Orbitrap were converted into MGF files by using Proteome Discoverer 1.2. The Mascot search engine, v2.3.02 (Matrix Science, London, United Kingdom), was adopted to identify the proteins on the NCBInr and SwissProt databases. The iTRAQ-identified proteins were matched to the GO,^7^ COG, and KEGG databases for protein functional annotation. A mass tolerance of 20 ppm was used for identifying intact peptides, whereas 0.05 Da was used for fragmented ions. Up to one missed cleavage in the trypsin digests was allowed. Only peptides correctly selected at 95% confidence interval based on Mascot probability were chosen to decrease the probability of false identification of peptides. Moreover, each confident protein identification involved at least one unique peptide. An intensity-based absolute quantification (iBAQ) algorithm was used to estimate the absolute protein abundance ranking (Schwanhäusser et al., 2011).

FASTA of bovine milk (Tacoma et al., 2016), sheep milk (Ha et al., 2015), swine milk (Ogawa et al., 2014), and human milk (Gao et al., 2012) was downloaded from UniprotKB^8^ to compare the protein ingredient of human milk with the milk of other mammals. Then, BLASTP was used to match the protein sequences of bovine milk, sheep milk, and swine milk (query argument) to human milk (subject argument). The alignment of BLASTP was based on an *E*-value of 1E-5 and identity ≥30%.

## Results

### Comprehensive Transcriptomic Analysis of 16 Donkey Tissues

Sixteen histologically healthy tissue specimens representing major donkey organs were analyzed by RNA sequencing (Table 1). Overall, 22,303 protein-coding genes and 68,480 transcripts were assembled from the mapped reads. A total of 6,290 novel genes and 24,514 novel transcripts were predicted, of which 1,365 were annotated by comparison with NR (621 novel genes), SwissProt (535 novel genes), eggNOG (922 novel genes), KEGG (151 novel genes), and Pfam (674 novel genes; intensity-based absolute quantification). Simultaneously, 16,013 protein-coding genes and 21,983 transcripts were successfully mapped to the reference genome. Further studies based on the reference genes were performed.

**FIGURE 1:**
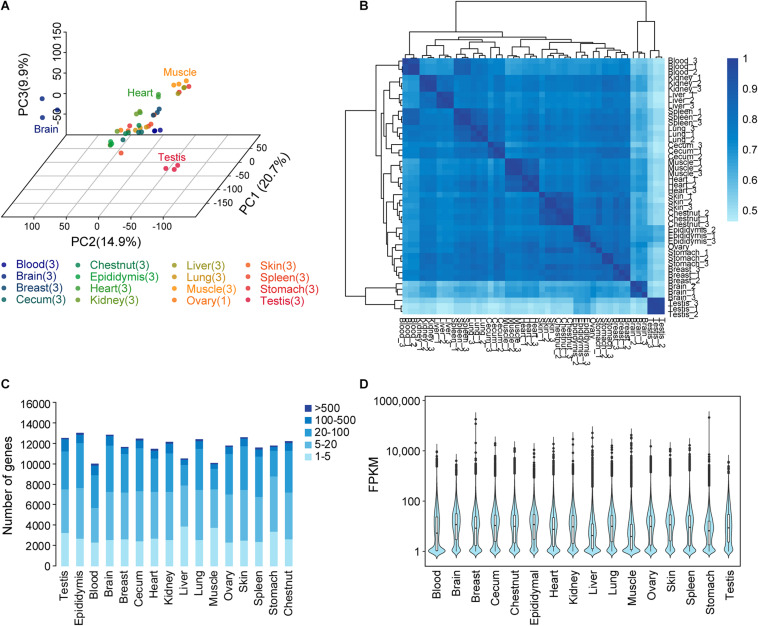
Donkey tissues and organs analyzed by transcriptome analysis. **(A)** Principal component analysis and sample location. The numbers in parentheses show the number of replicate samples for each tissue. Transcripts per million (TPM) was used as a measure of gene expression. **(B)** Relationship between tissues. Hierarchical clustering results showing the relationships between the 16 different tissues and organs and a heat map showing the pairwise Spearman correlation. TPM was used as a measure of gene expression. **(C)** Total number of genes with detected transcripts in each tissue using five different abundance levels for fragments per kilobase million (FPKM) values. **(D)** Transcript levels (FPKM) on a log10 scale for all genes for the 16 tissues.

The global expression profiles were investigated using PCA and hierarchical clustering on the basis of the correlation between 46 samples from 16 organs and tissues (Figures 1A,B). TPM was used as a measure of gene expression. The PCA revealed the testis and the brain as outliers, as also confirmed by hierarchical clustering analysis. Moreover, the hierarchical clustering analysis results uncovered the connectivity between the samples from the two striated muscle samples (cardiac and skeletal muscles), the hematopoietic organ spleen and blood, and the skin and chestnut.

A list of genes in each tissue is shown in Figure 1C. Gene classification was achieved on the basis of mRNA level (FPKM). The number of genes detected in the blood and epididymis averaged 10,035 and 12,987, respectively. The average FPKM for different tissues and organs ranged from 1 to >10,000 (Figure 1D).

### Classification of All Protein-Coding Genes Based on Transcriptomic Analysis

**FIGURE 2:**
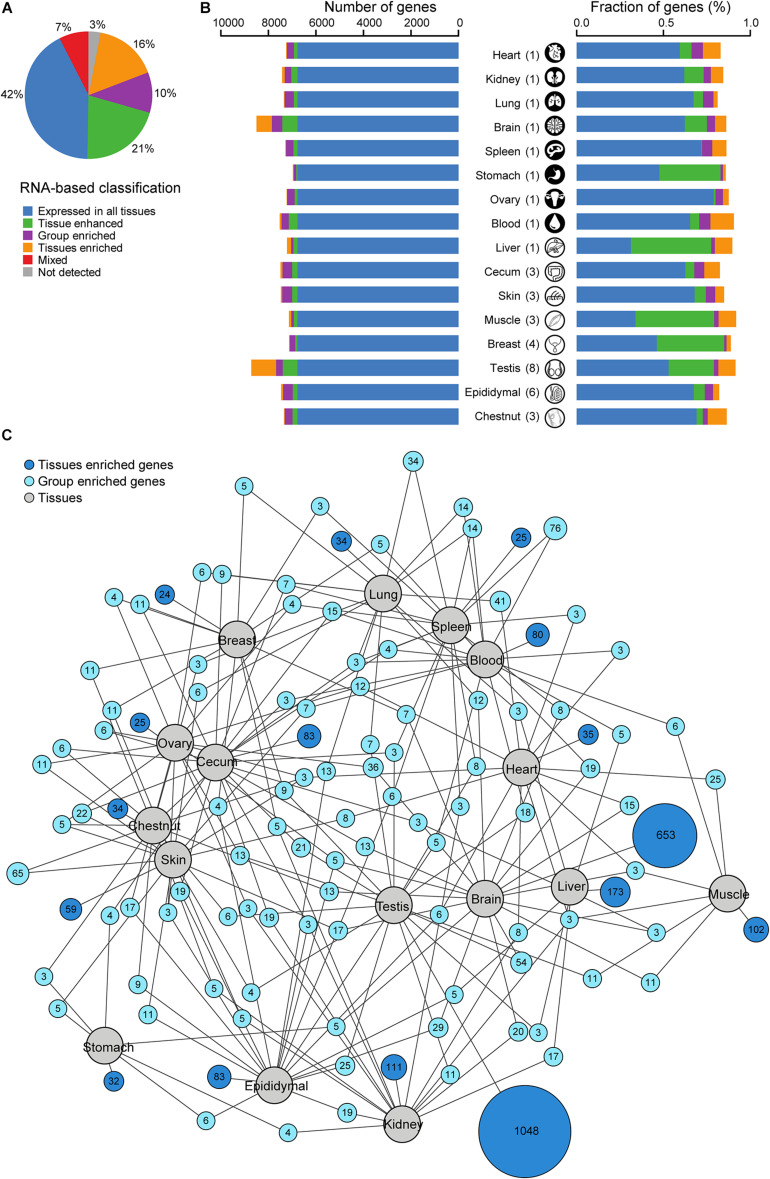
Classification of all donkey protein-coding genes with regards to the transcriptional levels in 16 tissues. **(A)** Pie chart showing the distribution of all 16,013 genes into six different categories based on the transcript abundance (fragments per kilobase million, FPKM). **(B)** Number and fraction of genes detected on the mRNA level in each tissue. FPKM was used as a measure of gene expression. The colors are the same as in panel **(A)**. **(C)** Network plot showing the relationship of tissue enriched and group enriched genes in various tissues and organs analyzed. Blue circle nodes represent a group of expressed genes and are connected to tissues represented by gray circles. Dark blue nodes show the number of group-enriched genes in up to five different tissue types (gray circles), with a minimum of three genes. Light blue nodes show the total number of tissue-enriched genes in each tissue. The size of each blue node represents the square root of the number of genes enriched in a particular combination of tissues. FPKM was used as a measure of gene expression.

All coding genes were classified as previously reported (Uhlén et al., 2015) to explore the proteins with a tissue-specific expression profile (Figure 2A and Supplementary Table 3). The average FPKM was used as a measure of gene expression. The largest group of genes (∼42%, *n* = 6,778/16,013) was expressed in all tissues. However, ∼47% (*n* = 7,559/16,013) of all genes were over-expressed in at least one tissue (“tissue-enriched,” “group-enriched,” or “tissue-enhanced”). The tissue-enriched genes constituted ∼16% (*n* = 2,601/16,013) of all genes, with ∼3% (*n* = 503/16,013) highly tissue-enriched with at least 50-fold over-expression of mRNA as exemplified by the breast enriched with α-lactalbumin (*LALBA*), the lung enriched with surfactant protein, and the brain enriched with myelin-associated oligodendrocyte basic protein. The group-enriched and tissue-enhanced genes comprised ∼10% (*n* = 1,627/16,013) and ∼21% (*n* = 3,331/16,013) of all genes, respectively. Furthermore, 3% of all genes (*n* = 502/16,013) were not detected in any tissues analyzed in this study.

Transcriptomic and network analyses (Figures 2B,C) revealed that only 1.0% (on average) of all genes showed a tissue-enriched profile. The brain (4.1%, *n* = 653/16,013) and the testis (6.5%, *n* = 1,048/16,013) were the notable exceptions, which displayed a substantially higher percentage of tissue-enriched genes. The transcriptomic analysis also allowed the determination of the fraction of elevated transcripts in each tissue. For the majority of tissues, ∼27% of the transcripts were encoded by tissue-elevated genes, except the muscle and the liver where the elevated genes encoded 58.0 and 58.4% of the transcripts, respectively. The network plot showed the number of tissue-enriched genes for each tissue and group-enriched genes shared with another tissue. The skin and chestnut shared many group-enriched genes as expected due to the similar cells consisting the tissues. As a hematopoietic organ, the spleen shared 76 group-enriched genes with blood. A notable detail is that the brain also shared several group-enriched genes with the gonads.

### Functional Analysis of Tissue-Enriched and Ubiquitously Expressed Genes

**FIGURE 3:**
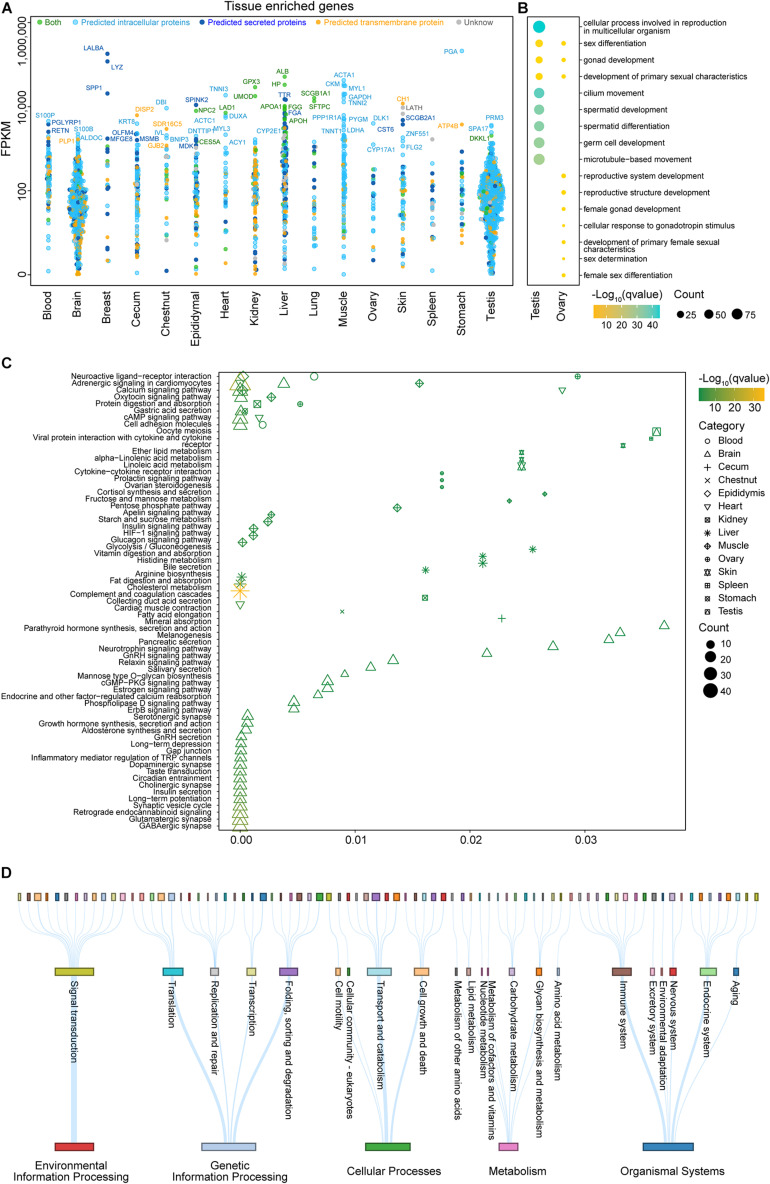
Analysis of tissue-enriched genes in different organ systems and ubiquitously expressed genes. **(A)** The transcript levels (fragments per kilobase million, FPKM) on a log10 scale for all genes identified as tissue-enriched are shown for 16 tissues, with each gene stratified according to the predicted localization. **(B)** An analysis of significant Gene Ontology (GO) terms (biological process) for the testis and ovary based on the tissue-enriched genes which were categorized on the basis of FPKM (for more details on the GO analysis, see Supplementary Table 4). **(C)** A Kyoto Encyclopedia of Genes and Genomes (KEGG) pathway analysis for all tissues based on the tissue-enriched genes which were categorized on the basis of FPKM (see Supplementary Table 5 for more details). **(D)** A KEGG pathway analysis for ubiquitously expressed genes which were categorized on the basis of FPKM. Each row represents a different KEGG level. The thickness of the lines and the sizes of the squares are proportional to the number of genes in the corresponding pathway (see Supplementary Table 6 for more details).

The functional categories and subcellular localizations of genes specifically expressed in certain tissues accurately reflected the long-standing function of tissues—for instance, many of the tissue-enriched genes of the testis were intracellular, while most tissue-enriched genes were secreted in the breast and the liver (Figure 3A). GO analysis revealed that the majority of testis-specific genes participated in spermatid development, differentiation, and motility (Figure 3B and Supplementary Table 4). The function of the testis to produce sperm was compatible with the subcellular localization of its tissue-enriched genes. By contrast, *LYZ* and *LALBA* encoding for secreted proteins LYSC1 and LALBA were specifically and highly expressed in the breast. Furthermore, the most abundant tissue-specific proteins for the liver were plasma proteins, e.g., albumin, haptoglobin, and many proteins that belonged to the cytochrome P450 superfamily of enzymes.

A functional KEGG pathway analysis for the tissue-enriched genes of 16 tissues is summarized in Figure 3C and Supplementary Table 5, and the results were consistent with the function of each tissue—for example, the heart-enriched genes encoded proteins that are involved in cardiac muscle contraction, the skin-enriched genes encoded proteins that are associated with lipid metabolism, and the brain-enriched genes were involved in functions related to signaling pathways and synapse. The brain-, heart-, and muscle-enriched genes were also observed to be involved in adrenergic signaling in cardiomyocytes and the calcium signaling pathway. The brain- and stomach-enriched genes participated in gastric acid secretion.

The transcriptomic analysis revealed that close to 7,000 genes (Supplementary Table 3) were expressed in all analyzed tissues. According to the result of the pathway analysis, these housekeeping gene regulates the structure and function of cellular organelles, such as transcription, translation, protein processing, proteolysis, transport, signal transduction, and energy metabolism (Figure 3D and Supplementary Table 6).

### Expression Levels of Lysozyme Gene Family in Different Tissues

**FIGURE 4:**
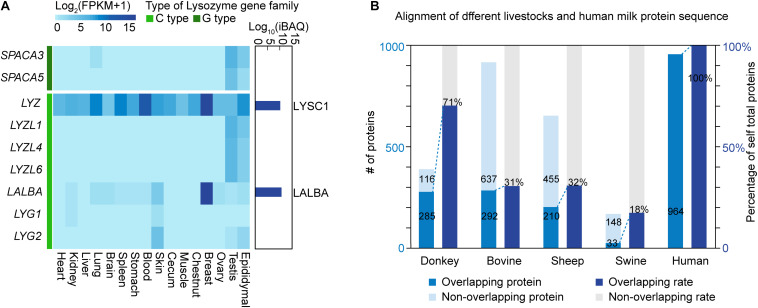
mRNA and protein expression levels of the lysozyme gene family. **(A)** RNA expression of the lysozyme family genes among tissues and protein expression of lysozyme family in donkey milk. The dark green bar represents goose-type lysozyme, and the green bar represents chicken or conventional-type lysozyme. The dark blue bars on the right represent log10-transformed intensity-based absolute quantification iBAQ values of lysozyme proteins in donkey milk. **(B)** Overlap analysis of milk proteins across species. The left *y*-axis represents the number of overlapping or non-overlapping proteins, and the right *y*-axis represents the proportion of overlapping proteins to total proteins.

In view of the high lysozyme content in donkey milk, *LYZ* and *LALBA* genes and their protein products were subsequently studied in depth. Seven c-type (chicken or conventional type; i.e., *LYZ*, *LALBA*, *LYZL1*, *LYZL4*, *LYZL6*, *SPACA5*, and *SPACA3*) and two g-type (goose type; i.e., *LYG1* and *LYG2*) lysozyme genes were detected to be expressed in different donkey tissues (Figure 4A). *LYZL1*, *LYZL4*, *LYZL6*, *SPACA5*, and *SPACA3* were highly expressed in the reproductive system, including the testis and the epididymis; *LYG1* and *LYG2* were highly expressed in the skin, and *LYZ* and *LALBA* were breast-enriched genes. Simultaneously, 265 proteins in donkey milk were quantified *via* the iBAQ method. The results showed that β-lactoglobulin-1, β-casein, LALBA, LYSC1, and κ-casein were the top five proteins with the highest content in donkey milk (Supplementary Table 7). According to protein annotation, *LYZ*, which encodes the LYSC1 protein, is an orthologous gene in donkey and horse. LYSC1 is a unique lysozyme protein to *Equus*. The IBQA analysis showed a high LYSC1 content in donkey milk. Furthermore, no other lysozyme proteins were found in donkey milk. The lysozyme concentration was 1.0 g/L in donkey milk, 0.79 g/L in mare milk, and 0.5 g/L in human milk, whereas it showed a trace amount in bovine, sheep, and swine milk (Table 2). Given that those intact *LYZ* genes that predict potentially functional LYSC1 were found in only a few species (e.g., donkey and horse), the high expression of *LYZ* in donkey breast may lead to a high lysozyme content in donkey milk.

**TABLE 2:** Concentration of lysozyme in milk from different species.

Species	Donkey	Mare^a^	Human^a^	Bovine^b^	Sheep^b^	Swine^c^
Lysozyme (g/L)	1.00	0.79	0.50	Traces	Traces	Traces

*^*a*^Miranda et al. (2004). ^*b*^Polidori and Vincenzetti (2013). ^*c*^Lu et al. (2015).*

Moreover, a total of 401 non-redundant proteins in donkey milk were identified by the iTRAQ method (Table 3 and Supplementary Figure 1), of which 381 were annotated by comparison with GO (235 proteins), COG (182 proteins), and KEGG (373 proteins) databases (Supplementary Table 8 and Supplementary Figures 2–4). In particular, the KEGG pathway enrichment analysis revealed that proteins were mainly enriched in immune and inflammatory response-related pathways, such as complement and coagulation cascades, lysosome, and phagosome. After a BLASTP search was conducted, 285 proteins in donkey milk, representing 71% of total self-proteins, were found to overlap with human milk proteins. Moreover, the number of overlapping proteins in bovine, sheep, and swine milk was 292 (representing 31% of total self-proteins), 210 (32%), and 33 (18%), respectively, (Figure 4B).

**TABLE 3:** Statistics of the basic information on the identification of donkey milk protein.

Sample	Total spectra	Spectra	Unique spetra	Peptide	Unique peptide	iTRAQ Protein	iBAQ Protein	Annotation		
								GO	GOC	KEGG
Donkey milk	298,419	20,759	20,314	1,242	1,169	401	265	235	182	373

## Discussion

In this study, a tissue-based map of the donkey genome was presented from the analyses of 16 tissues, with gene expression data on the RNA level and supplementary analyses on the protein level for additional donkey milk samples. A total of 16,013 putative protein-coding genes were identified and stratified into six classes: “expressed in all,” “tissue-enriched,” “group-enriched,” “tissue-enhanced,” “mixed,” and “not detected.” Approximately 6,700 genes across all donkey tissues regulated housekeeping processes, including cell growth, signal transduction, and metabolic pathways. Around 2,600 genes displayed a tissue-enriched expression pattern across all major organ systems. A functional analysis of tissue-specific genes revealed that the tissue performed the long-established roles. In addition, 401 non-redundant proteins were identified, with 265 quantified in donkey milk. Notably, *LYZ* and *LALBA* were specifically and highly expressed in the breast, and so are their encoding proteins LYSC1 and LALBA in donkey milk. Furthermore, 71% of the donkey milk proteins overlapped with human milk protein, showing the highest overlapping rate among bovine, sheep, and swine.

The transcriptome results in donkey were compared with those in other mammals. The genome, transcriptome, and proteome of human are the best characterized among all species. Wang et al. (2019) and Uhlén et al. (2015) detected ∼18,000 protein-coding genes with an average of 12,000 genes per tissue in humans by RNA sequencing. Whole-genome gene expression arrays detected 17,924 protein-coding genes in mouse (Su et al., 2004). One cattle transcriptome study showed that 16,564 genes were detected by analyzing 100 different tissues and cell types (Liu et al., 2020). In addition, approximately 18,528, 19,921, and 14,426 protein-coding genes were detected in domestic goat (Muriuki et al., 2019), sheep (Clark et al., 2017), and pig (Freeman et al., 2012), respectively. The number of detected genes in donkey is equivalent to that in cattle. Moreover, 6,290 novel genes were detected in the present study. Overall, a transcriptome atlas of 16 donkey tissues was obtained.

In addition, the brain and the testis exhibited a higher percentage of tissue-enriched proteins, which were also found in human (Uhlén et al., 2015; Wang et al., 2019), cattle (Liu et al., 2020), sheep (Clark et al., 2017), and goat (Muriuki et al., 2019), than other organs. The result of the functional analysis showed that brain-enriched genes were significantly associated with synapse and neuron function and testis-enriched genes with spermatogenesis and reproduction in humans (Wang et al., 2019), cattle (Liu et al., 2020), and donkey. Due to the high specificity of function, the expression of these genes may be restricted to spermatogenic cells or neurons. The expression of a tissue-enriched gene was conserved across these mammals, and so were the housekeeping genes. Identified by the same method, approximately 6,700 and 9,000 ubiquitously expressed housekeeping genes were observed in donkey and humans (Uhlén et al., 2015), respectively. The housekeeping genes identified by a cluster analysis in sheep (Clark et al., 2017), goat (Muriuki et al., 2019), and pig (Freeman et al., 2012) were also clustered into several largest clusters. Housekeeping genes are fundamental to maintain essential cellular structure and function.

The lysozyme is a critical, non-specific immune protein in human milk, and it plays the role of a shield against bacterial invasion. The lysozyme concentration is higher in donkey milk than in human and horse milk, but it has trace amounts in bovine, sheep, and swine milk. Guo et al. (2007) also found a high lysozyme content in donkey milk. *LYZ* expressed at high levels in the breast and encoded LYSC1 protein in horses and donkeys. However, it was highly expressed in the rumen and encoded digestive lysozyme rather than LYSC1 in ruminants, such as cow and sheep (Callewaert and Michiels, 2010). The high expression of the horse orthologs of the donkey gene, *LYZ*, in donkey breast may contribute to the high LYSC1 content in donkey milk. The high concentration of lysozyme is one of the benefits of donkey milk as a surrogate for breast milk (Conte and Panebianco, 2019). Another advantage of donkey milk is its similar chemical composition with human milk (Souroullas et al., 2018). The casein components and antigen in the whey proteins of donkey milk are more closely related to human homologs than their cow counterparts (Barłowska et al., 2011). The donkey milk proteomic profile in the present study also showed that 71% of the proteins in donkey milk overlapped with human milk protein, higher than the overlapping rates of bovine, sheep, and swine with humans. As a substitute for human milk, donkey milk should be studied more adequately.

The gene expression atlas for donkey tissues presented here represents a much improved transcriptome for donkey. Similarly, the donkey milk proteomic profile yields valuable qualitative and quantitative protein information. We detail the gene expression profile of 16 tissues and protein expression in donkey milk, complementing the existing *E. asinus* genome annotations and contributing to the available genomic resources to interpret the molecular mechanisms underlying phenotype traits. By utilizing transcriptomics and proteome, *LYZ* and *LALBA* were found to be specifically and highly expressed in donkey breast in the present study, and so are their encoding proteins LYSC1 and LALBA in donkey milk. *LYZ* and LYSC1 were connected, thus laying a foundation for further study of the regulatory mechanism of high lysozyme expression. Other inter-species comparison studies between humans and livestock could significantly enhance the utility of genomic, transcriptomic, and proteomic information and facilitate the exploration of the relationship between genotype and phenotype.

## Data Availability Statement

The datasets presented in this study can be found in online repositories. The names of the repository/repositories and accession number(s) can be found below: https://www.ncbi.nlm.nih.gov/genbank/, PRJNA431818 and https://www.ncbi.nlm.nih.gov/genbank/, PRJNA498718.

## Ethics Statement

The animal study was reviewed and approved by the Institutional Animal Care and Use Committee of Shandong Academy of Agricultural Sciences.

## Author Contributions

SL and YiW designed the study. YiW, XM, and ZZ carried out the data acquisition and analysis. YiW, SL, and CW wrote the manuscript. XM, ZZ, and YoW collected the samples and managed the data. YiW and ZZ contributed to the bioinformatics analysis and figure creation. YoW contributed to the table preparation. CW and SL supervised the study. All authors read and approved the final manuscript.

## Conflict of Interest

ZZ was employed by the company Shenzhen Byoryn Technology Co., Ltd. The remaining authors declare that the research was conducted in the absence of any commercial or financial relationships that could be construed as a potential conflict of interest.

## Publisher’s Note

All claims expressed in this article are solely those of the authors and do not necessarily represent those of their affiliated organizations, or those of the publisher, the editors and the reviewers. Any product that may be evaluated in this article, or claim that may be made by its manufacturer, is not guaranteed or endorsed by the publisher.

## Footnotes

1
www.fao.org/faostat/en/#home

2
www.ncbi.nlm.nih.gov/protein

3
www.uniprot.org

4
eggnog.embl.de

5
www.kegg.jp

6
pfam.xfam.org

7
geneontology.org

8
http://www.uniprot.org/help/uniprotkb

## Supplementary Material

Supplementary Figure 1 The distribution of the identified protein in donkey milk.

Supplementary Figure 2 Gene Ontology annotation for all identified proteins.

Supplementary Figure 3 Clusters of Orthologous Groups annotation for all identified proteins.

Supplementary Figure 4 Kyoto Encyclopedia of Genes and Genomes pathway annotation for all identified proteins.

Supplementary Table 1 Tissue sample information.

Supplementary Table 2 Annotation of novel genes compared with NR, SwissProt, eggNOG, Kyoto Encyclopedia of Genes and Genomes, and Pfam databases.

Supplementary Table 3 Average fragments per kilobase million values for genes identified in 16 tissue transcriptomes and their tissue-specific classification.

Supplementary Table 4 Functional Gene Ontology analysis for ovary and testis.

Supplementary Table 5 Functional Kyoto Encyclopedia of Genes and Genomes pathway analysis for tissue-enriched genes of 16 tissues.

Supplementary Table 6 Functional Kyoto Encyclopedia of Genes and Genomes pathway analysis for ubiquitously expressed genes.

Supplementary Table 7 Intensity-based absolute quantification values of the 265 identified proteins in donkey milk.

Supplementary Table 8 Annotation of proteins compared with Gene Ontology, Clusters of Orthologous Groups, and Kyoto Encyclopedia of Genes and Genomes databases.
