# Supplementary material for: Transcriptome Atlas of 16 Donkey Tissues
Source: Front Genet. 2021 Aug 9;12:682734. doi: 10.3389/fgene.2021.682734 (PMC8381363; doi:10.3389/fgene.2021.682734)
Supplement: Supplementary Figure 1 — The distribution of the identified protein in donkey milk. [file Data_Sheet_2.docx]

# Supplementary Figures


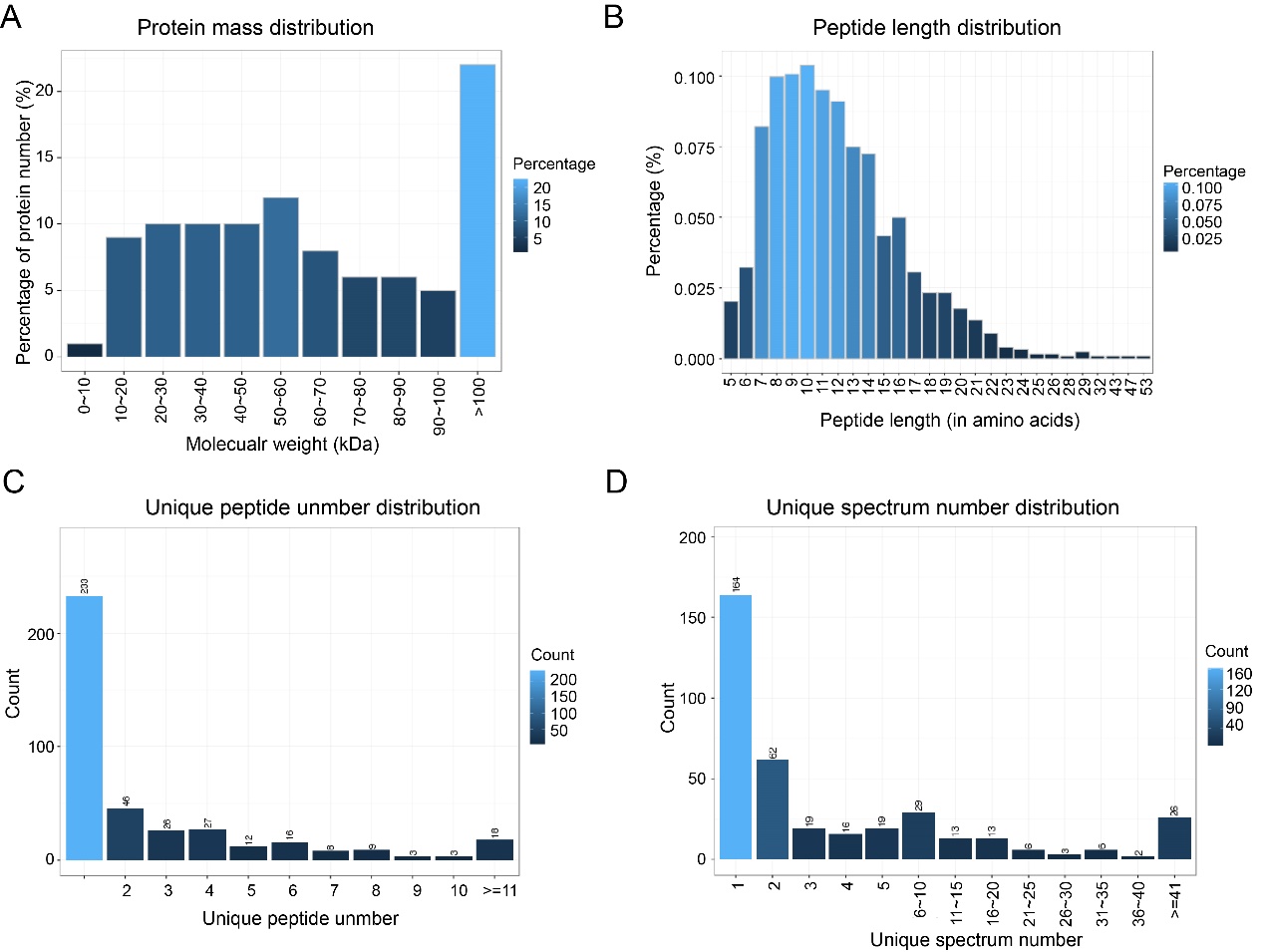


**Figure. S1** The distribution of identified protein in donkey milk. (A) Protein mass distribution. X-axis: Molecular weight(kDa); Y-axis: Percentage of protein number. (B) Peptide length distribution. This picture depicts the percentage of different peptide lengths. X-axis: the peptide length; Y-axis: the corresponding peptide percentage. (C) Unique peptide number distribution. The X-axis shows the unique peptide number of each protein and the Y-axis shows the corresponding protein number. The trend of this picture describes that most of the identified proteins contain less than 10 peptides and protein quantity become less with the increase of peptide. (D) Unique spectra number distribution. This picture is similar to (C). The X-axis shows the unique spectrum number of each protein and the Y-axis shows the corresponding protein number.


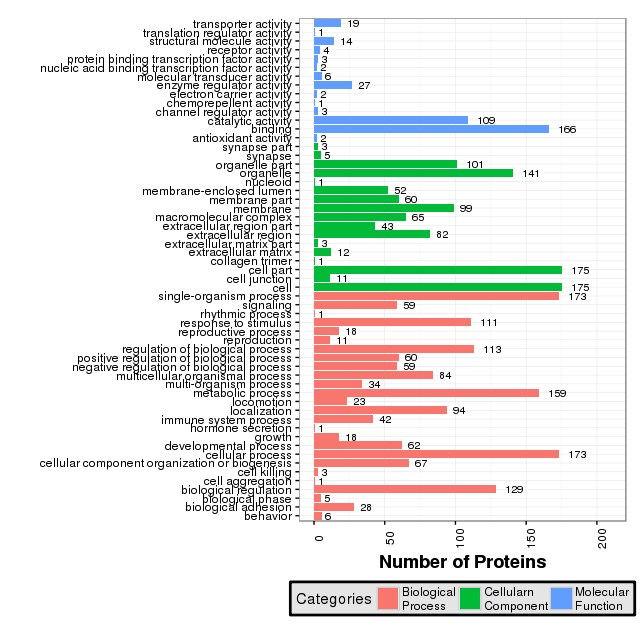


**Figure. S2** GO annotation for all identified proteins. The X-axis displays the corresponding protein count illustrating the protein number of different functions, and the y-axis displays the GO term.


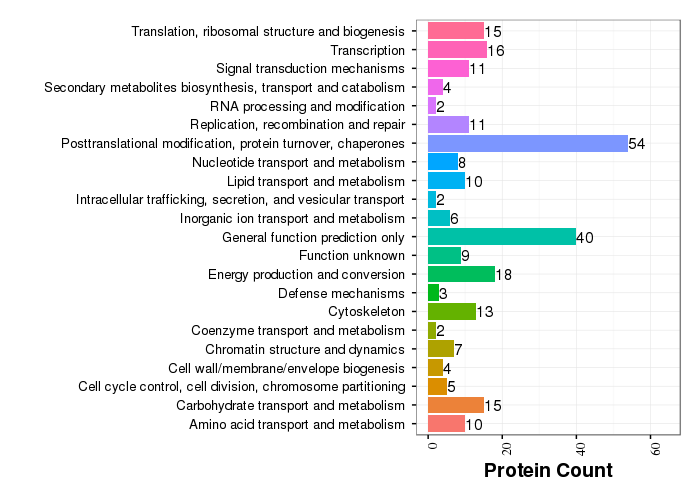


**Figure S3** COG annotation for all identified proteins. The X-axis displays the corresponding protein count illustrating the protein number of different functions, and the y-axis displays the COG term.


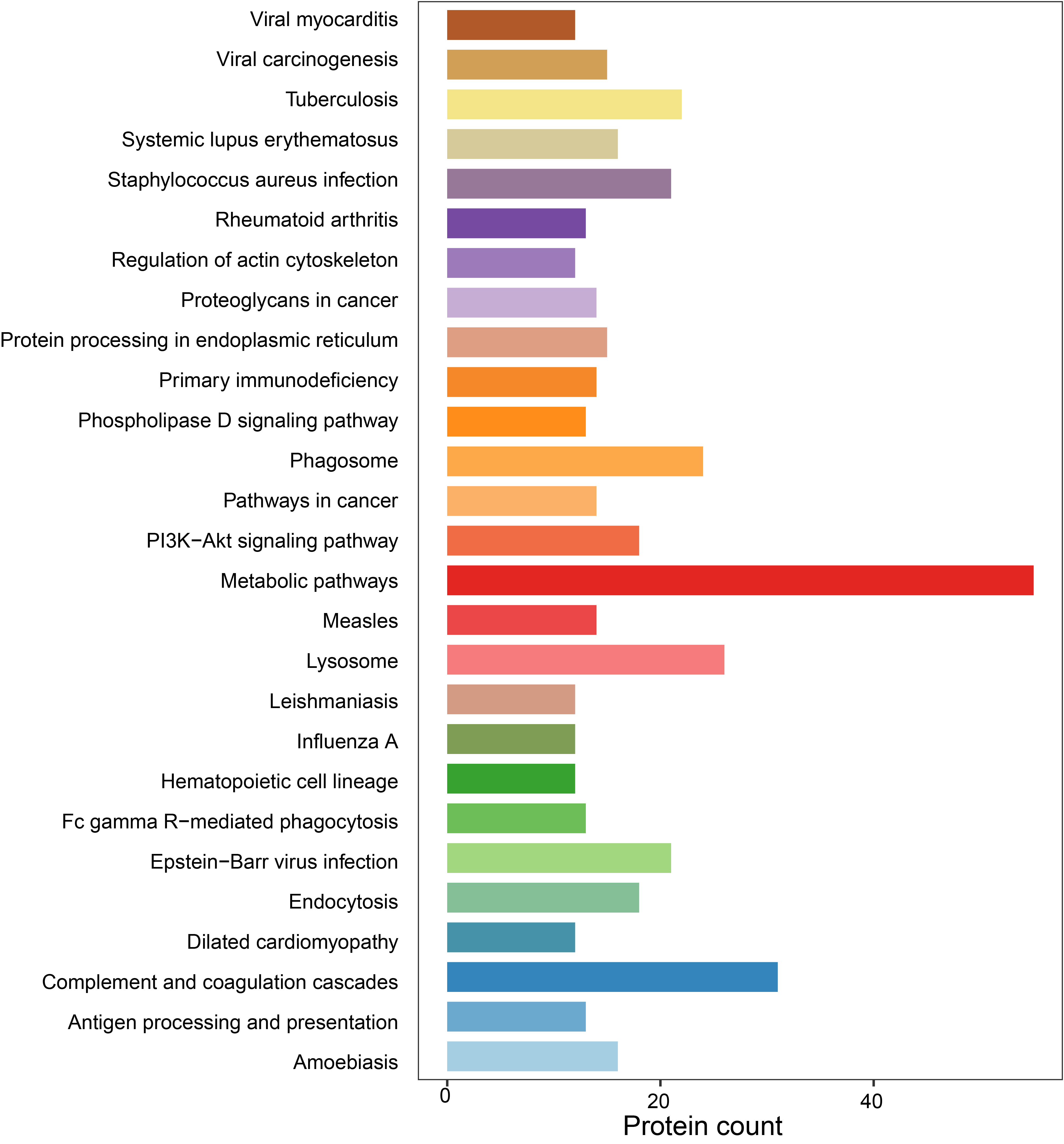


**Figure S4** KEGG pathway annotation for all identified proteins. The X-axis displays the corresponding protein count illustrating the protein number of different functions, and the y-axis displays the KEGG pathways (protein count > 12).
